# The effectiveness of preoperative assessment using a patient-specific three-dimensional pseudoarticulation model for minimally invasive posterior resection in a patient with Bertolotti’s syndrome: a case report

**DOI:** 10.1186/s13256-020-02635-y

**Published:** 2021-02-16

**Authors:** Kensuke Shinonara, Michiya Kaneko, Ryo Ugawa, Shinya Arataki, Kazuhiro Takeuchi

**Affiliations:** Department of Orthopaedic Surgery, Okayama Medical Center, National Hospital Organization, 1711-1 Tamasu, Kitaku, Okayama city, Okayama Japan

**Keywords:** Bertolotti’s syndrome, Intraoperative three-dimensional navigation, Minimally invasive resection, Three-dimensional sterical plaster model

## Abstract

**Background:**

Bertolotti’s syndrome is widely known to cause low back pain in young patients and must be considered as a differential diagnosis. Its treatment such as conservative therapy or surgery remains controversial. Surgical procedure is recommended for intractable low back pain. The three-dimensional (3D) lumbosacral transitional vertebrae anatomy should be completely understood for a successful surgery. Using an intraoperative 3D navigation and preoperative preliminary surgical planning with a patient-specific 3D plaster model contribute for safe surgery and good outcome.

**Case presentation:**

A case of a 22-year-old Japanese male patient with intractable left low back pain due to lumbosacral transitional vertebrae with Bertolotti’s syndrome. The symptom resisted the conservative treatment, and anesthetic injection at pseudoarticulation only provided a short-term pain relief. Posterior resection using intraoperative three-dimensional (3D) navigation has been performed through microendoscopic view. Pseudoarticulation was totally and successfully resected in a safe manner.

**Conclusions:**

Preoperative surgical planning and rehearsal using a patient-specific 3D plaster model was greatly useful and effective for surgeons in performing accurate and safe pseudoarticulation resection.

## Introduction

Bertolotti’s syndrome is characterized by congenital lumbosacral transitional vertebrae (LSTV) most commonly occurring at L5 level and by enlarged transverse process of L5 articulates or unilateral or bilateral unions with the sacral area [1]. This syndrome has been reported to result in low back pain in young patients [2].

It is typically treated with conservative therapy, such as medication, trigger point anesthetic injection, and rehabilitation. In cases of intractable low back pain, surgical intervention is considered [3–7]. Several procedures for LSTV resection have already been reported; however, performing accurate resection remains difficult due to the congenital bone defect. The three-dimensional (3D) LSTV anatomy should be completely understood for a successful surgery. Here, we present the postoperative outcome of minimally invasive LSTV resection using an intraoperative 3D navigation and the effectiveness of preoperative preliminary surgical planning using a patient-specific 3D plaster model of LSTV. The institutional review board approved this clinical case report (Okayama Medical Center, Number: 2018-181).

## Case presentation

**Fig. 1:**
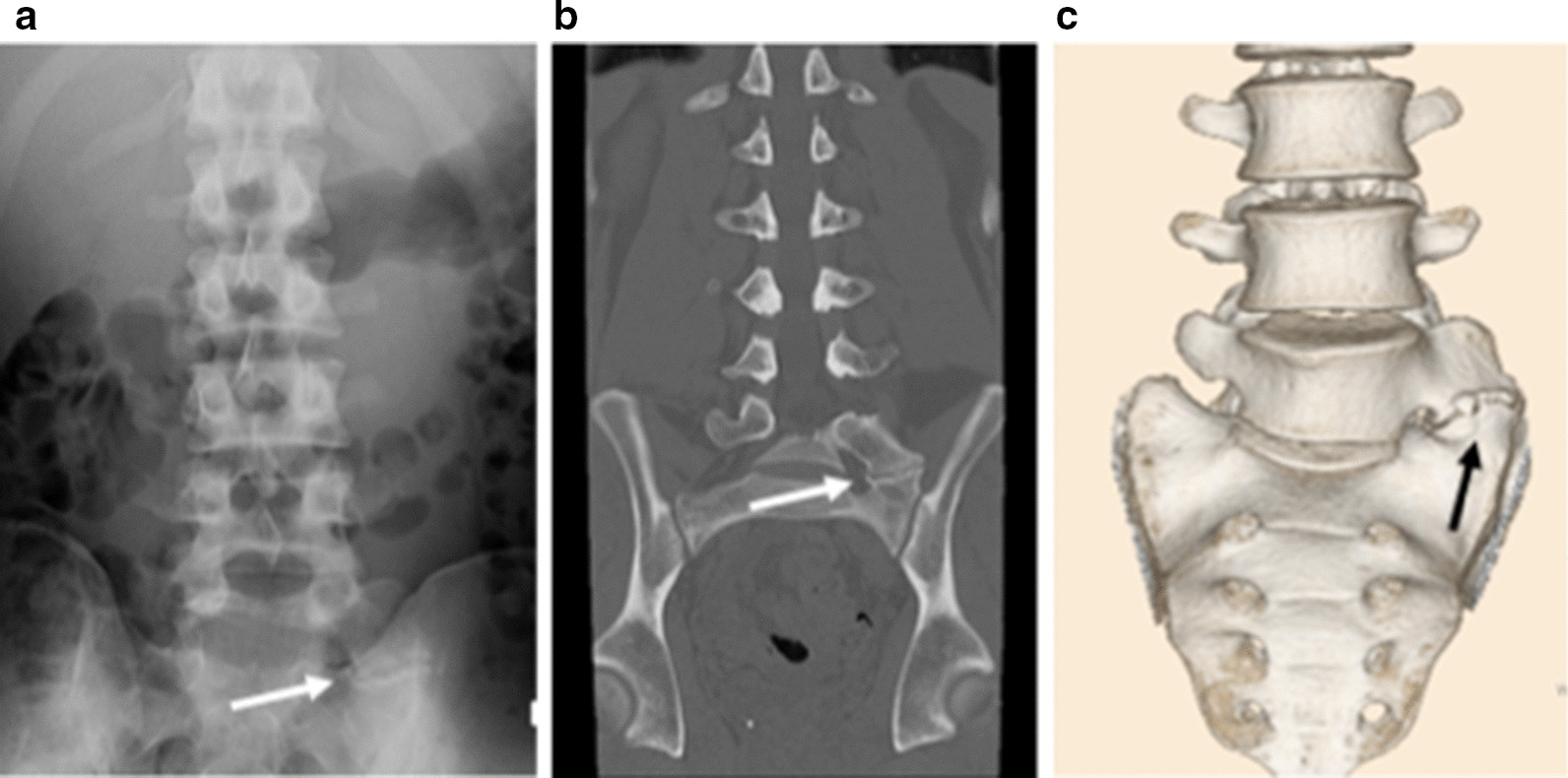
**a** Preoperative anteroposterior plain radiography. **b** Preoperative coronal computed tomography (CT) image. **c** Preoperative three-dimensional (3D) CT image. Arrow indicates pseudoarticulation at the left side.

**Fig. 2:**
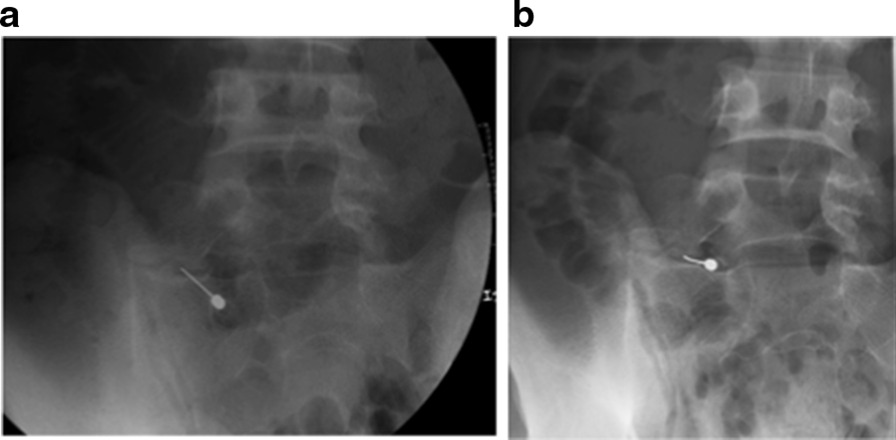
Anesthetic injection during pseudoarticulation

**Fig. 3:**
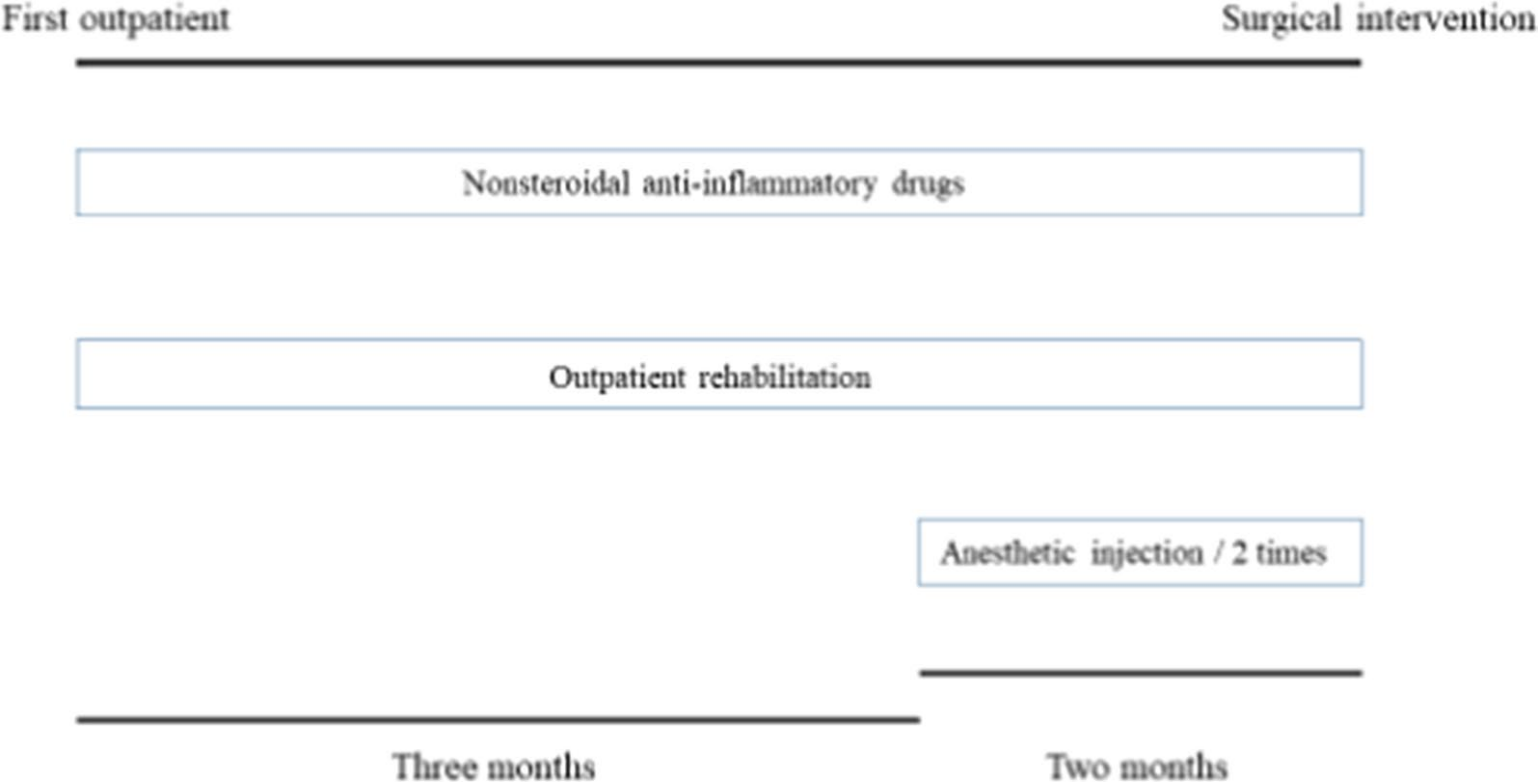
The diagram shows the timeline of conservative treatment

A 22-year-old Japanese man who worked as carpenter complained of chronic left low back pain for at least 6 months. He had no remarkable medical history and traumatic event for a long time. His physical examination showed no neurological deficits, such as muscle weakness and sensory changes. In the assessment of physical status, Oswestry Disability Index (ODI) [8] was 46%. Radiographs and computed tomography (CT) images showed the LSTV at the L5 level, identified as Castellvi classification type 2A [9] (Fig 1). MRI revealed no degenerative disc changes at L5–S1 level and no compression at the left L5 exiting nerve root. Conservative treatment with non-steroidal anti-inflammatory drugs and rehabilitation was immediately initiated in accordance with the therapeutic strategy reported by Li et al for first three months [10]. Anesthetic injection at the LSTV pseudoarticulation was performed twice during next two months (Fig. 2) and provided temporary pain relief every time. However, long-term improvement was not achieved. Conservative treatment was performed for total five months (Fig. 3).

After the recurrence of left low back pain, a minimally invasive tubular surgery was recommended.

### Surgical procedure

**Fig. 4:**
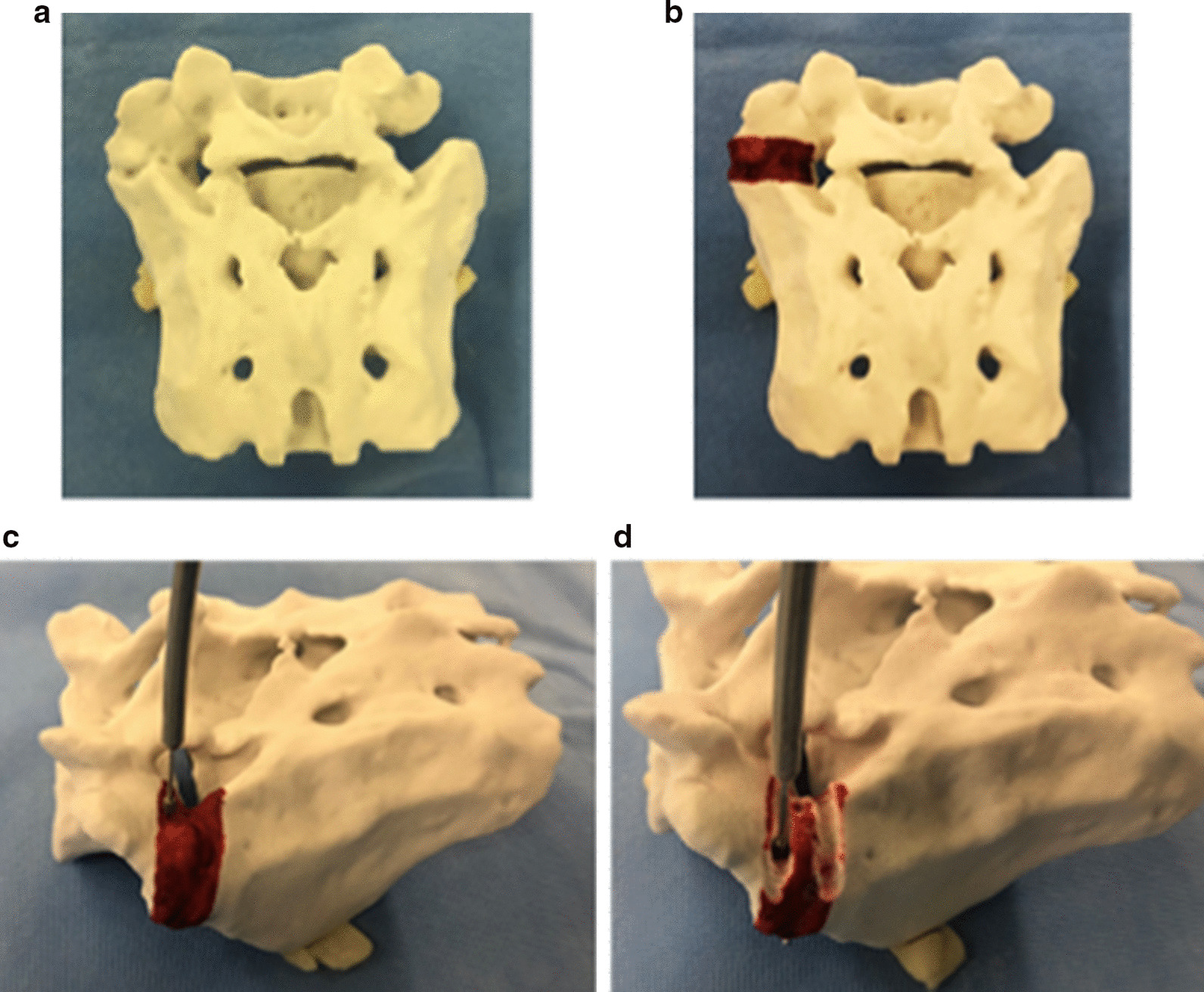
**a** Three-dimensional plaster model of the left lumbosacral transitional vertebrae. **b** Painted area shows the range to be resected. **c**, **d** Rehearsal of resection using a high-speed drill

Preoperatively, a 3D plaster model (Biotec Bones, Zimmer Biomet KK, Tokyo) of LSTV was produced using a 3D printer to sterically grasp and decide the area to be resected and was also used for training and rehearsal of the surgical procedure (Fig. 4).

**Fig. 5:**
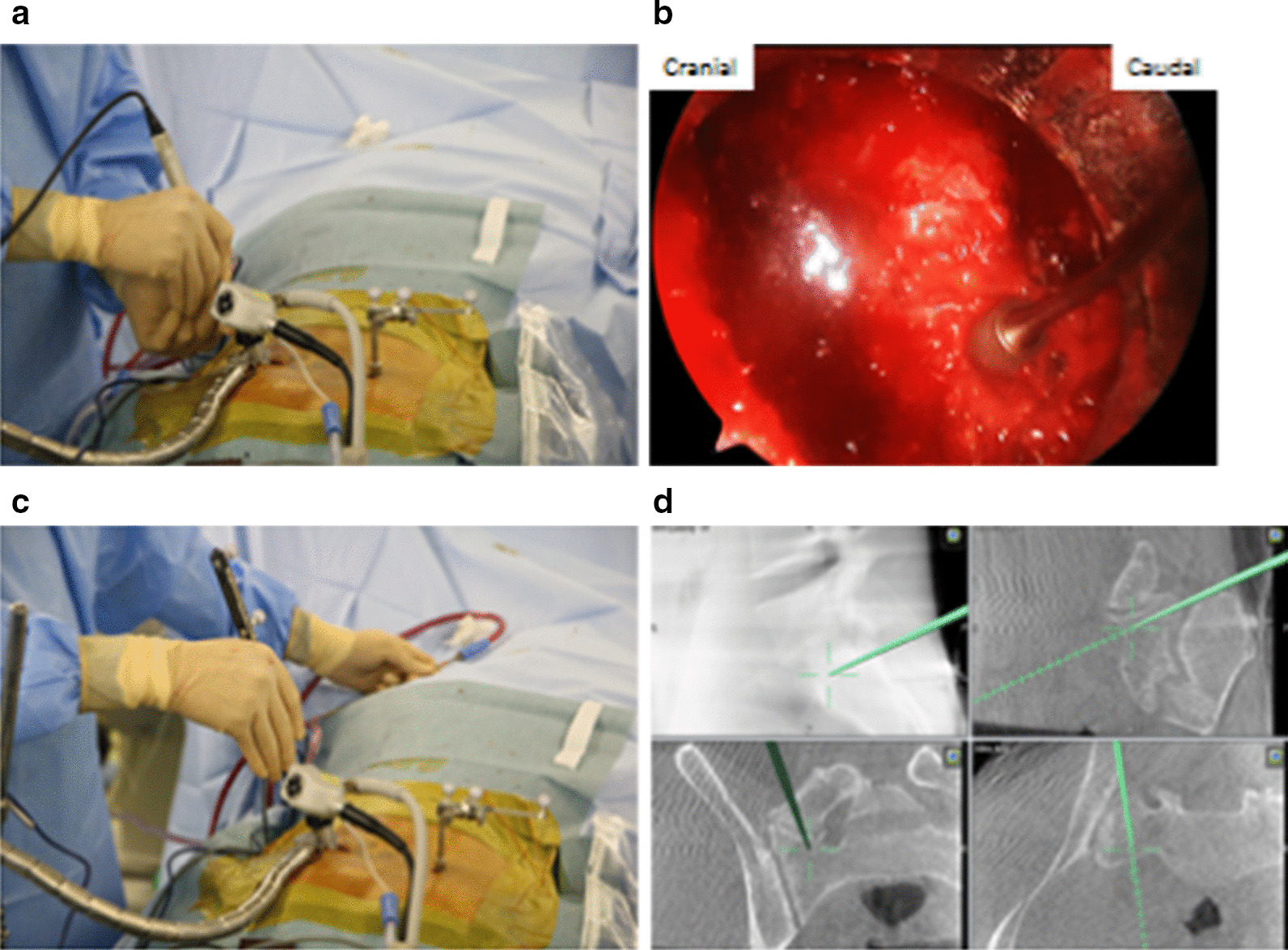
**a** Placement of microendoscope at the left side and navigation of the referential array in the right iliac crest placement. Resection using a high-speed drill. **b** Intraoperative microendoscopic image. Resection of pseudoarticulation using a high-speed drill. **c** Checking the depth and progress status of excavation (real-time images are shown in **d**). **d** Intraoperative three-dimensional navigation images

**Fig. 6.**
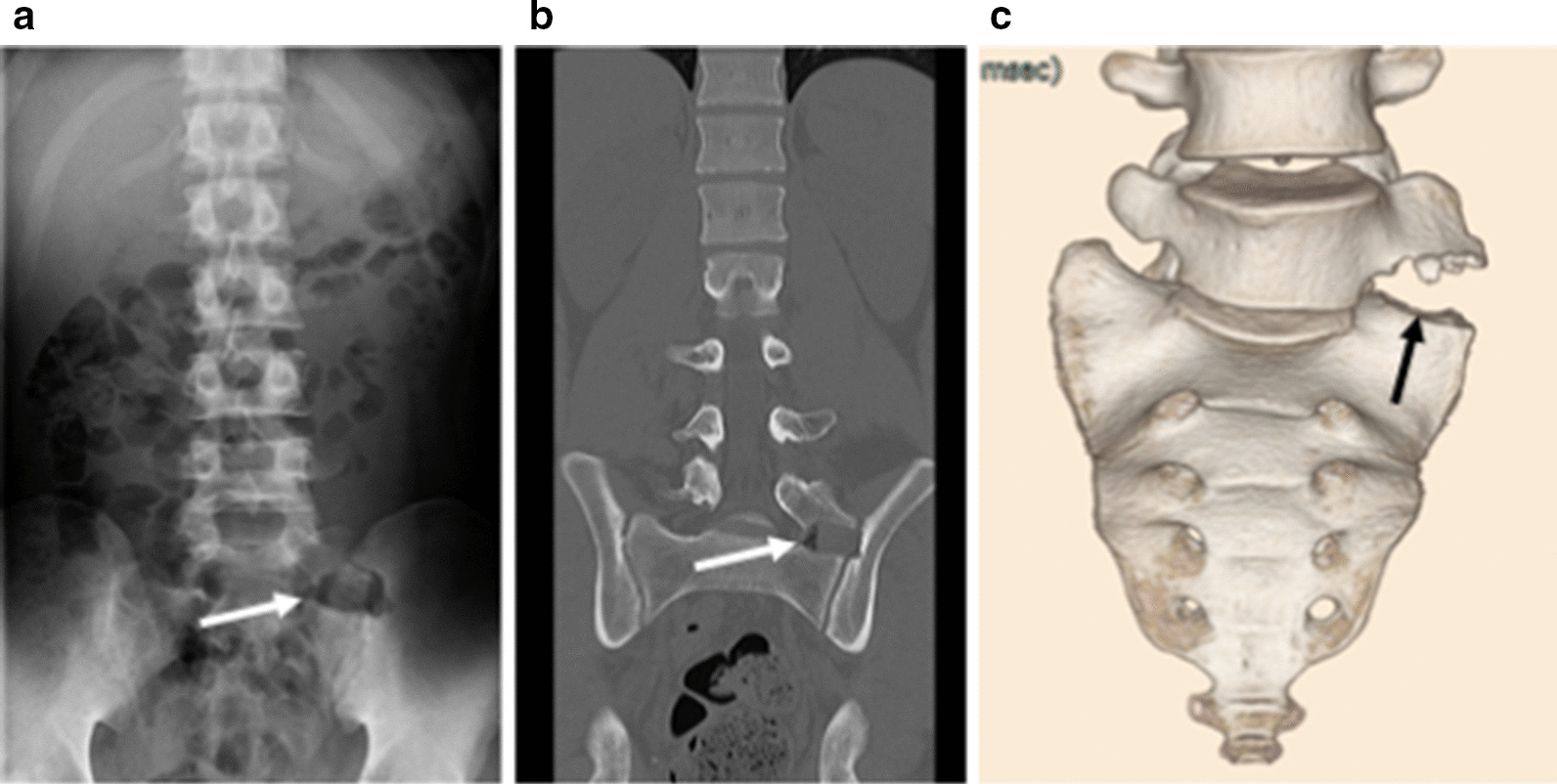
**a** Postoperative anteroposterior plain radiography. **b** Postoperative coronal computed tomography (CT) image. **c** Postoperative three-dimensional (3D) CT image. Arrow indicates the removal of pseudoarticulation

The patient was placed in prone position on the operating table under general anesthesia. The navigation referential array was inserted into the right posterior iliac crest, and intraoperative images were obtained using a C-arm (Siemens, Germany) and navigation system (Brainlab, Germany). A 4.0-cm transverse incision was made over the LSTV at the left side, and the left posterior iliac crest was resected to facilitate a direct approach to the psuedoarticulation. A 1.8-mm microendoscopic tubular portal was placed strictly at the operating table, and the LSTV was resected using high-speed drill, bone chisel, and Kerrison punch. The tubular portal was moved side to side during each resection. We sometimes checked the progress status of excavation using a navigation probe (Fig. 5). After the complete resection, the left iliac crest bone was returned to its original position and fixated using a fiber wire. No intraoperative and postoperative complications occurred. Postoperative radiographs and CT images showed adequate LSTV resection (Fig. 6). The patient was free from the left low back pain, and the asymptomatic status was maintained at 2 months follow up. Postoperative ODI was 6%.

## Discussion

Bertolotti’s syndrome was reported by Bertolotti nearly one century ago [5]. This syndrome is characterized by chronic low back pain with or without radiculopathy. Quinlan *et al.* reported that the overall incidence of Bertolotti’s syndrome was 4.6% and that in young patients aged < 30 years was 11.4% [2]. However, it is commonly misdiagnosed as low back pain in young patients [11].

Castellvi *et al.* classified LSTV of Bertolotti’s syndrome into four types [9], and Jancuska *et al.* reported types 2 and 4 as highly positively related with low back pain [12].

Although no consensus has been reached on its treatment, conservative therapy has been recommended as the first choice. Diagnostic algorithm and treatment strategy were described by Li *et al.* [10]. Even recently, several researchers have reported surgical options; however, the appropriate surgical method remains controversial. Although many literatures reported the use and outcome of surgical posterior resection [1, 4, 5, 10], this is the first report that used a 3D plaster model of LSTV for surgical planning and strategy identification preoperatively. The effectiveness of patient-specific 3D plaster model is to understand the details of LSTV sterically and rehearse the methods.

Intraoperative 3D navigation images helped surgeons determine the progress and depth of excavation. Additionally, navigation images will promote performance of safe surgery because the major vessels and exiting nerve roots are placed around the LSVT. Babu *et al.* reported that intraoperative 3D images and navigation guidance facilitated resection of enlarged transverse processes [13]. Therefore, the use of intraoperative 3D navigation is recommended for a safe and complete LSTV resection.

In this case, a minimally invasive posterior surgery was chosen for LSTV resection because no degenerative disc changes were observed at the L5–S1 level. In young patients, minimally invasive surgery can effectively reduce postoperative changes. If the degenerative disc change is detected, fusion surgery is required.

## Conclusions

Berotolotti’s syndrome is effectively diagnosed not only by radiographs and CT images but also by anesthetic injections at pseudoarticulation. Patients with intractable low back pain associated with Bertolotti’s syndrome that resisted conservative therapy can be effectively treated with minimally invasive posterior resection of LSTV. Therefore, preoperative surgical planning with a patient-specific 3D plaster model and intraoperative navigation images are suggested to be useful for surgeons, which result in a safe surgery and complete LSTV resection.

## Data Availability

Not applicable.

## Abbreviations

LSVT: Lumbosacral transitional vertebrae
3D: Three-dimensional
ODI: Oswestry Disability Index
CT: Computed tomography

## References

[1] Louie CE, Hong J, Bauer DF. Surgical management of Bertolotti’s syndrome in two adolescents and literature review. Surg Neurol Int. 2019;10:135. 10.25259/SNI-305-2019 PMC6744759 31528470

[2] Quinlan JF, Duke D, Eustace S. Bertolotti’s syndrome. A cause of back pain in young people. J bone Joint Surg Br. 2006;88:1183–6. 10.1302/0301-620X.88B9.17211 16943469

[3] Malham GM, Limb RJ, Claydon MH, et al. Anterior pseudoarthrectomy for symptomatic Bertolotti’s syndrome. J Clin Neurosci. 2013;20:1762–6. 10.1016/j.jocn.2013.02.018 23969004

[4] Takata Y, Sasaki T, Higashino K, et al. Minimally invasive microendoscopic resection of the transverse process for treatment of low back pain with Bertolotti’s syndrome. Case Rep Orthop. 2014; 613971 10.1155/2014/613971 PMC4089204 25045566

[5] Yousif S, Wood M. Minimally invasive resection of lumbosacral pseudojoint resulting in complete resolution of a lower back pain—a case report and review of Bertolotti’s syndrome. J Clin Neurosci. 2018;51:67–8. 10.1016/j.jocn.2018.02.006 29503029

[6] Adams R, Nicol SH, Jenkins A. Surgical treatment of a rare presentation of Bertolotti’s syndrome from Castellvi type IV lumbosacral transitional vertebra: case report and review of literature. J Neurol Surg Rep. 2018;79:e70–4. 10.1055/s-0038-1667172 PMC6066366 30083494

[7] Santavirta S, Tallroth K, Ylien P, et al. Surgical treatment of Bertolotti’s syndrome. Follow-up of 16 patients. Arch Orthop Trauma Surg. 1993;112:82–7. 10.1007/BF00420261 8457417

[8] Fujiwara A, Kobayashi N, Saiki K, et al. Association of the Japanese Orthpaedic Association score with the Oswestry Disability Index, Roland-Morris Disability Questionnaire, and short-form 36. Spine. 2003;28:1601–7. 12865852

[9] Castellvi AE, Goldstein LA, Chan DP. Lumbosacral transitional vertebrae and their relationship with lumbar extradural defects. Spine. 1984;9:493–5. 10.1097/00007632-198407000-00014 6495013

[10] Li Y, Lubelski D, Abdullah KG, et al. Minimally invasive tubular resection of the anomalous transverse process in patients with Bertolotti’s syndrome. J Neurosurg Spine. 2014;20:283–90. 10.3171/2013.11.SPINE13132 24358999

[11] Manmohan S, Dzulkarnain A, Nor Azlin ZA, et al. Bertolotti’s syndrome: a commonly missed cause of back pain in young patients. Malays Fam Physician. 2015;10:55–8. PMC4826583 27099663

[12] Jancuska JM, Spivak JM, Bendo JM, et al. A review of symptomatic lumbosacral transitional vertebrae: Bertolotti’s syndrome. Int J Spine Surg. 2015;9:42. 10.14444/2042 PMC4603258 26484005

[13] Babu H, Lagman C, Kim TT, et al. Intraoperative navigation-guided resection of anomalous transverse processes in patient with Bertolotti’s syndrome. Surg Neurol Int. 2017;8:236. 10.4103/sni.sni_173_17 PMC5629866 29026672

